# Epstein-Barr virus, human papillomavirus and herpes simplex virus 2 co-presence severely dysregulates miRNA expression

**DOI:** 10.4102/ajlm.v10i1.975

**Published:** 2021-03-16

**Authors:** Jude O. Okoye, Anthony A. Ngokere, Charles C. Onyenekwe, Olaposi Omotuyi, Deborah I. Dada

**Affiliations:** 1 Department of Medical Laboratory Science, School of Public and Allied Health, Babcock University, Ilishan-Remo, Ogun State, Nigeria; 2 Department of Medical Laboratory Science, Faculty of Health Sciences and Technology, College of Medicine, Nnamdi Azikiwe University, Nnewi campus, Anambra, Nigeria; 3 Department of Biochemistry, Centre for Biotechnology, Adekunle Ajasin University, Akungba-Akoko, Ondo, Nigeria

**Keywords:** Epstein-Barr virus, human papilloma virus, herpes simplex virus 2, epithelial-mesenchymal transition, microRNAs

## Abstract

This cross-sectional study evaluated the expression of miR-let-7b, miR-21, miR-125b, miR-143, miR-145, miR-155, miR-182, miR-200c, p53 gene, Ki67, SCCA1 and CD4+ T-cell counts among 319 women, to Epstein-Barr virus, human papillomavirus and herpes simplex virus 2 mono-infections and co-infections, using enzyme-linked immunosorbent assay and reverse transcriptase-polymerase chain reaction methods. This study suggests that malignancies associated with viral co-infection could be diagnosed early by monitoring cluster of differentiation 4+ T-cell counts and serum expression of miR-145 and miR-182.

## Introduction

Evidence shows that Epstein-Barr virus (EBV) infection favours human papillomavirus (HPV) fusion to host genome resulting in genetic instability, initiation and amplification of epithelial-mesenchymal transition (EMT) to cancer in the host.^1,2^ The mechanisms by which they synergistically promote cancer invasiveness and metastasis are yet to be fully understood. Although controversies still exist concerning the role of type 2 herpes simplex virus (HSV-2) in cervical carcinogenesis, mounting evidence suggests that the persistence and reactivation of HSV-2 not only facilitates EBV and HPV infection entry but also increases the risk of cervical squamous cell carcinoma by 60 times.^3,4^ This viral co-infection may elicit diverse, overwhelming dysregulation of the host’s oncomirs-cancer-associated micro-ribonucleic acids (miRNAs) or the tumour suppressor miRNAs. MicroRNA is a short unit of non-coding RNA that post-transcriptionally regulates gene expression.^5^ Viruses are known to cause overexpression or underexpression of certain miRNA, especially in individuals with cancer.^5^ In this study, we ascertained whether the extent of miRNA overexpression or underexpression is higher or lower in viral co-infection than in mono-infection, and which type of co-infection is associated with the highest and lowest expression of miRNA when compared with uninfected individuals. Hence, this study evaluated the expression of normally downregulated biomarkers (miR-21, miR-155, miR-182, miR-200, p53 gene, Ki67, and squamous cell carcinoma antigen 1 [SCCA1]) and normally upregulated biomarkers (miR-let-7b, miR-125b, miR-143, miR-145, and CD4+ T-cells) in individuals with single through ternary viral infections and those without the targeted viral infections (EBV, HPV and HSV-2). Overexpressed and underexpressed miRNAs could serve as biomarkers for identifying and or monitoring individuals who are at risk of developing cervical lesions.^6^

## Methods

### Ethical considerations

For this study, ethical approvals were obtained from the State Hospital Abeokuta Ethics Committee (SHA/RES/VOL.2/177) and Babcock University Health Research Committee (BUHREC549/18). Written consent was obtained from all participants. The questionnaires filled by the participants and samples taken following selection were given specific numbers in place of names to protect the privacy of participants. Personal information and results of the participants remained undisclosed to other personnel, except the investigators and respective participants.

### Study area and participants

This cross-sectional study was carried out between May 2017 and August 2018 during a free screening programme. Using the convenience sampling technique, this study included active commercial sex workers (*N* = 319; mean age = 33.84 ± 8.38 years) living in selected brothels in Abeokuta and Ilishan-Remo communities. Interested participants visited the family planning clinic at their own expense. Sex workers who voluntarily visited the family planning clinic were selected by researchers based on the description provided by the reports of the United States Centers for Disease Control and Prevention, the United Nations for AIDS and World Health Organization.^7,8^ The demographics were collected using an interview-based questionnaire. Inclusion criteria included being a self-proclaimed direct (commercial; receive money for sex) and sexually active female sex workers aged 18–54 years. Individuals with any history of cervical, breast and oral cancers or those that are transgender or below 18 years or above 54 years were excluded.

### Sample collection and handling

The majority of samples were taken at the Family Planning Unit, State Hospital Ijaiye, Abeokuta and the Phlebotomy Unit, Babcock University Teaching Hospital, Nigeria, by nurses and researchers. Some samples were collected at the brothels only on the insistence of concerned participants. All samples (blood and Pap smears) were collected according to the standard operating procedure of the hospitals. Participants were screened for cervical cancer using liquid base preparation and Papanicolaou techniques. Smears were taken by nurses and clinicians and evaluated by two cytopathologists. Five millilitres (mL) of peripheral blood was drawn between the hours of 09:00 and 10:00, from each participant, by Medical Laboratory Scientists; 3 mL was drawn into an ethylenediaminetetraacetic acid vacutainer tube for CD4+ T-cell counts and 2 mL was drawn into a plain tube for enzyme-linked immunosorbent assay (ELISA). Samples taken at the Abeokuta collection centre were transported to the Chemical Pathology Unit, Babcock University Teaching Hospital, in a specimen transport cooler containing some ice packs. All samples were analysed at the Chemical Pathology Unit, Babcock University Teaching Hospital. After allowing the blood in the plain tube (2 mL) to stand for not more than 2 h, clear serum was separated into another plain tube and stored at −20 °C until analysed. CD4+ T-cell counts and Pap smears were investigated daily while other test were carried out at intervals (≤ 2 months).

### Sample assays

The CD4 easy count kit and Cyflow Counter (Sysmex Partec GmbH, Gorlitz, Germany) were used for counting CD4+ T-cells in whole blood, not more than 2 h after collection. To maintain CD4^+^ T-cell day-to-day counts, two internal quality controls were employed: previously tested whole blood samples and stabilised whole blood samples. The separated sera were tested for antibodies against HIV-2, EBV, HPV, and HSV-2 by ELISA (Qingdao Hightop Biotech Co. Ltd, Qingdao, Shandong, China, and Calbiotech Inc., El Cajon, California, United States) according to manufacturers’ instructions. According to the manufacturers instructions, a 96% to 99% agreement (performance) was observed between ELISA kits and the reference ELISA method. Serum samples from participants whose cervical cells had HPV and HSV-2 viral cytopathic effects –koilocytes and viral cytoplasmic inclusions– were considered as internal positive controls. Previously tested samples from laboratory and clinically confirmed HIV positive and negative cases were used as internal positive and negative controls for HIV testing. P16 regulates the cell cycle by acting as a cyclin-dependent kinase inhibitor and has been used as an alternative biomarker for HPV testing.^10,11^ Thus, serum samples that were positive and negative for p16 (from previous a study) were also used as internal positive and negative controls for HPV testing. p16 kit-included positive and negative controls were considered as external controls (Bioassay Technology Laboratory, Yangpu, Shangai, China). The cut-off values for positive and negative controls were ≥ 8100 ng/L and ≥ 8099 ng/L, respectively. Both internal and external positive and negative controls were used in the calibration and calculation of cut-off values for HPV and HSV-2 antibodies. Furthermore, serum samples from cervical cancer patients whose cervical tissues or biopsies tested positive for EBV (LMP-1), HPV and HSV-2 antigen by immunohistochemistry were used as internal positive controls while those whose cervical tissues or biopsies tested negative for the viral antigens by immunohistochemistry were used as internal negative controls (using a commercial kit from Bio SB Inc., Santa Barbara, California, United States). In all four positive and four negative controls (based on koilocyte presence, p16 positivity, HPV immunohistochemistry status and manufacturer’s HPV control sample) were used for HPV testing while three positive and three negative controls (based on viral inclusion in cervical cells, HSV-2 immunohistochemistry status, and manufacturer’s HSV-2 control sample) were used for HSV-2 testing. More so, two positive and two negative controls (based on EBV immunohistochemistry status and manufacturer’s EBV control sample) were used for EBV testing. The controls were included during assay (in order to eliminate false positives and forestall false negatives), prior to calculation of cut-offs. The sera were also tested by the ELISA method for human proliferation-related ki-67 antigen (Ki67; using kits from Melsin Medical Co. Ltd, Changchun, Jilin, China) and squamous cell carcinoma antigen 1 (SCCA1; using kits from Elabscience Biotechnology Co. Ltd, Wuhan, Hubei, China). Using the internal and external control samples, tests for viral antibodies were considered valid when the mean negative control optical density was 0.1 or lower and the mean positive control optical density was 0.8 or higher. Based on the formula provided by the manufacturers of the ELISA kits, test samples were considered positive when their viral antibody estimates were greater than the cut-off values. The positive cut-off values for HIV, HPV IgG, HPV IgM, HSV-2 IgG, HSV-2 IgM, EBV IgG, and EBV IgM antibodies were 1.077, 1.071, 0.438, 1.520, 0.102, 1.570 and 1.030 or higher, respectively, while the negative cut-off values for the parameters were 1.076, 1.070, 0.438, 1.519, 0.101, 1.569 and 1.029 or lower, respectively. The positive cut-off values for Ki67 and SCCA1 were 7849 ng/mL and 1670 pg/mL or higher while the negative cut-off values were 7848 ng/mL and 1669 pg/mL or lower.

### Gene expression profiling

#### RNA isolation and complementary DNA synthesis

Using the optimised phenol-chloroform RNA extraction method,^12^ 50 *µ*L of serum was added to Eppendorf tubes containing 50 *µ*L trizol reagent and vortexed at 2500 revolutions per minute for 15 min. Chloroform (100 *µ*L) was added to the mixture which was subsequently vortexed and centrifuged for 30 min at 1500 revolutions per minute. The supernatants containing the RNA were aspirated into new labelled tubes. Iso-amyl alcohol (100 *µ*L) was added to the supernatant with subsequent centrifugation for 30 min at 1500 revolutions per minute. The RNA was recovered in pellet form following decantation of the supernatant. The RNA pellet was washed thrice with 70% ethanol. Fifty *µ*L of 70% ethanol was added to the tube and the tube was centrifuged for 5 min at 1500 revolutions per minute. After which, the supernatant was decanted. After washing all tubes were allowed to air dry. Fifty microlitre (*µ*L) of nuclease-free water was added to the total RNA to form the RNA solution. The total RNA concentration (48 *µ*L of deionised distilled water + 2 *µ*L of RNA solution) was quantified spectrophotometrically at 260 nanometre. The RNA limit of importance was set at 0.05–1.00. The complementary DNA synthesis was carried out by adding miRNA-universal stem-loop primer cocktail (containing nuclease-free water, the reverse transcriptase buffer, the reverse transcriptase, miRNA- universal stem-loop primer specific oligos, and oligo deoxyribonucleotide triphosphate) to 20 *µ*L of total RNA of each sample. The samples were then incubated at room temperature overnight. The concentration of complementary DNA was determined spectrophotometrically at 260 nanometre and homogeneity was carried out on every sample.

#### Reverse transcriptase polymerase chain reaction

The amplification was performed using optimisation and the universal stem-loop primer method.^13^ In order to validate our primers for miRNA quantification listed in Table 1, we test ran the primers against samples from known cervical cancer patients (as a positive control; *n* = 10) and patients with normal cervix (negative control; *n* = 10). The expression of oncomirs were at least fourfold higher in cancer patients than in healthy individuals while the expression of tumour suppressors were at least threefold higher in healthy individuals than in cancer patients. Template (complementary DNA, 2 *µ*L), nuclease-free water (3 *µ*L), forward primer (0.5 *µ*L) and reverse primer (0.5 *µ*L; Inqaba Biotechnical Industries Ltd, Hatfield, Pretoria, South Africa) and master mix (4 *µ*L; New England Biolabs GmbH, Frankfurt, Germany) were added to each sample in no specific order. All reactions were done as singleplexes for each biomarker. Amplification conditions were: 94 °C pre-denaturation for 5 min, 94 °C for 30 s, annealing 55 °C for 30 s and extension 72 °C for 30 s and then 5 min at 72 °C by 45 cycles.

**TABLE 1:** List of primer sequences.

miRNA/RNA	Direction	Primer sequence	Source	Amplicon size E.N.C.
hsa-miR-let-7b	Forward	5’-GTTTCGGGGTGAGGTAGTA-3’	This study	131 bp
hsa-miR-16	Forward	5’-GTTGTCAGCAGTGCCTTAG-3’	This study	68 bp
hsa-miR-21	Forward	5’-GGTGTCGGGTAGCTTATCA-3’	This study	22 bp
hsa-miR-125b	Forward	5’-GTTTTGCGCTC CTCTCAGT-3’	This study	105 bp
hsa-miR-143	Forward	5’-TTTTTGCGCAGCGCCCTG-3’	This study	115 bp
hsa-miR-145	Forward	5’-GTTTCACCTTGTCCTCACG-3’	This study	110 bp
hsa-miR-155	Forward	5’-GTTTCTGTTAATGCTAATCGTGATA-3’	This study	23 bp
hsa-miR-182	Forward	5’-GTTTTAGA ACTCACACGTGTGA-3’	This study	24 bp
hsa-miR-200c	Forward	5’-GTTTCCCTCGTCTTACCCA-3’	This study	31 bp
Universal reverse primer	-	5’-GTGCAGGGTCCGAGGT-3’	^14^	-
Universal stem-loop primer	-	5’-AGTGCAGGGTCCGAGGTATTCGCAC CAGAGCCAACATGTCACG-3’	This study	-
p53	Forward	5’-GCTCAAGACTGGCGCTAAAA-3’	-	-
-	Reverse	5’-GTGACTCAGAGAGGACTCAT-3’	^15^	43 bp
β-actin	Forward	5’-ACACTTTCTACAATGAGCTGCG-3’	-	-
-	Reverse	5’-ACCAGAGGCATACAGGACAAC-3’	^15^	97 bp

miRNA, microRNA; E.N.C.; expected in normal condition; bp, base pairs.

#### Gel electrophoresis

**FIGURE 1:**
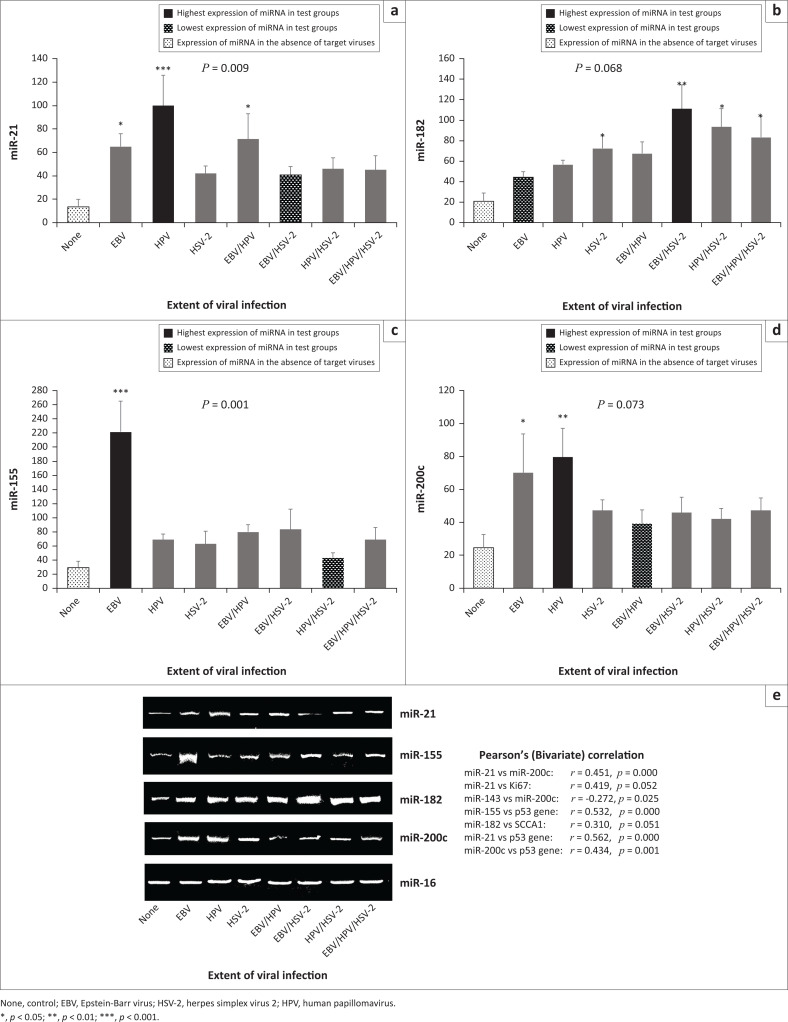
Relative expression of oncogenic miRNAs among individuals with and without viral infection, Ogun State, Nigeria, 2018. (a) miR-21 expression was higher among participants with HPV mono-infection; (b) miR-182 expression was higher among participants with EBV and HSV-2 bi-infection; (c) miR-155 expression was higher among participants with EBV mono-infection; (d) miR-200c expression was higher among participants with HPV mono-infection; (e) Snapshots of gel amplicons of oncomirs and normalising gene (miR-16) based on viral status (plotted as a–d).

**FIGURE 2:**
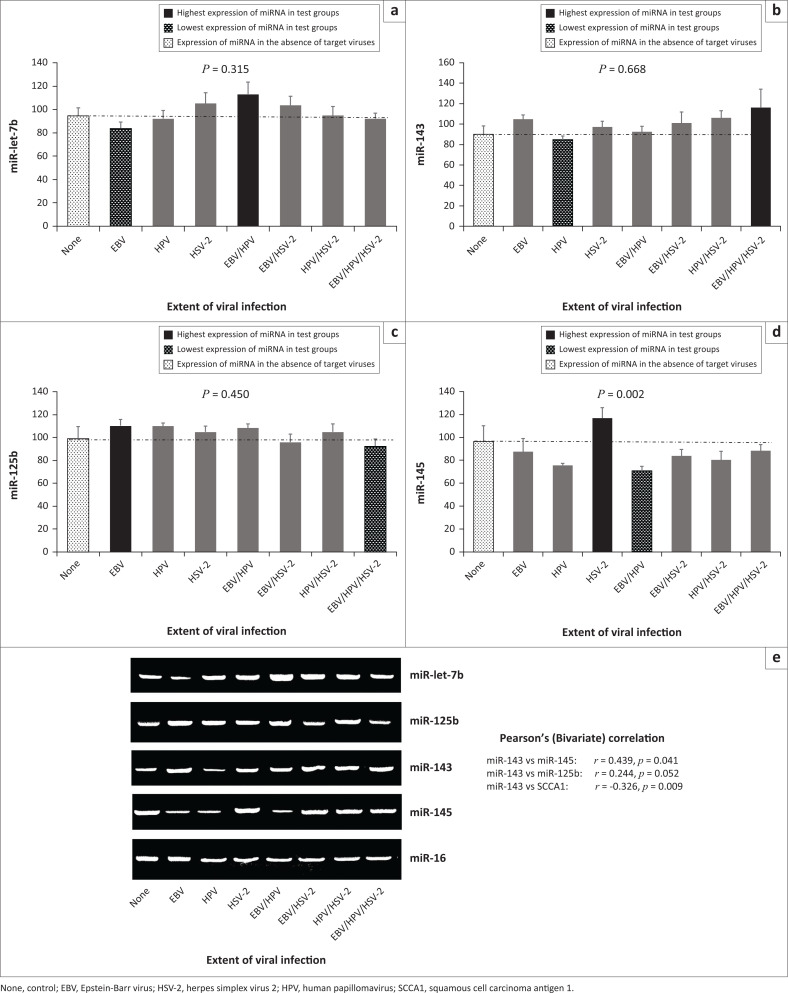
Relative expression of tumour suppressor miRNAs in individuals with and without viral infection, Ogun State, Nigeria, 2018. (a) miR-let-7b expression was higher among participants with EBV and HPV bi-infection and lower among participants with EBV mono-infection; (b) miR-143 expression was higher among participants with EBV, HPV and HSV-2 tri-infection and lower among participants with HPV mono-infection; (c) miR-125b expression was higher among participants with EBV mono-infection and lower among participants with EBV, HPV and HSV-2 tri-infection; (d) miR-145 expression was higher among participants with HSV-2 mono-infection and lower among participants with EBV and HPV bi-infection (d); (e) snapshots of gel amplicons of tumour suppressors and normalising gene (miR-16) based on viral status (plotted as a–d).

**FIGURE 3:**
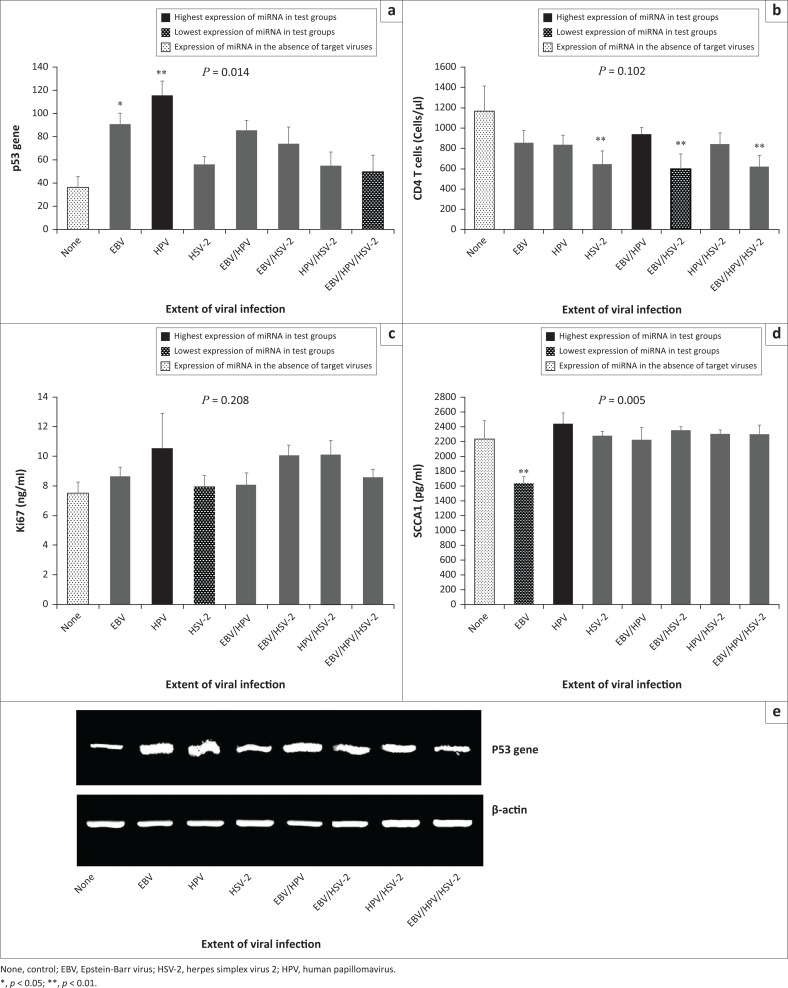
Biomarker levels and CD4+ T-cell counts among individuals with and without viral infection, Ogun State, Nigeria, 2018. (a) p53 gene expression was higher among participants with HPV mono-infection and lowest in control group, participants who had no viral infections (None); (b) CD4+ T-cell counts were higher among the control group (None) and lower among those with EBV and HSV-2 co-infection; (c) Ki67 expression was higher among participants with HPV infection and lower in the control (None); (d) SCCA1 expression was higher among participants with HPV mono-infection and lower in those with EBV mono-infection; (e) snapshots of gel amplicons of p53 gene and normalising gene (β-actin) based on viral status (plotted as a).

Products from polymerase chain reaction (PCR) were electrophoresed in 0.5% of agarose gel using 0.5× Tris-borate-ethylenediaminetetraacetic acid buffer of pH 8.3 (2.6 g of Tris base, 5.0 g of Tris boric acid and 2 mL of 0.5 M ethylenediaminetetraacetic acid; Thermo Fisher Scientific Johannesburg Pty, Germiston, South Africa) with 0.2 *µ*L ethidium as a fluorescent tag. The expression products were visualised as bands (amplicons) using an ultraviolet-transilluminator. To minimise image variations, the camera of an iPhone (set at 10 cm above the surface of the transilluminator and without flash or zoom; 326 pixels per inch) was used in acquiring snapshots of the amplicons (Figures 1e, 2e and 3e). Densitometrical analysis of the snapshots was carried out using ImageJ software (version 1.49; National Institute of Mental Health, Bethesda, Maryland, United States). Using the ImageJ software adjustment tool, the snapshots were scanned and background impurities (including non-ethidium bromide luminescence and other reflections from gel particles) were eliminated. Only the full area of the bands (specific ethidium bromide luminescence) was selected and quantified by the software. Results were expressed in figures; three decimal places. Relative expression of miRNAs and the p53 gene were calculated following endogenous normalisation using miR-16 for miRNAs and *β*-actin for the p53 gene.^16^

### Statistical analyses

Descriptive statistics were carried out to categorise participants into the eight groups: Group 1 (None): EBV, HPV and HSV-2 seronegative participants; Group 2: EBV mono-infection; Group 3: HPV mono-infection; Group 4: HSV-2 mono-infection; Group 5: EBV/HPV bi-infection; Group 6: EBV/HSV-2 bi-infection; Group 7: HPV+HSV-2 bi-infection; and Group 8: EBV/HPV/HSV-2 tri-infection.

The relative expression of miRNAs and the p53 gene in the healthy control group (uninfected commercial sex workers) and in the different viral status groups were compared using analysis of variance, partial correlation and Pearson’s (bivariate) correlation in Statistical Package for Social Sciences (version 21; IBM Corporation, Armonk, New York, United States). In terms of viral status (coded as ‘0’ for no infection, ‘1’ for EBV mono-infection, ‘2’ for HPV mono-infection and ‘3’ for HSV-2 mono-infection, ‘4’ for EBV and HPV bi-infection, ‘5’ for EBV and HSV-2 bi-infection, ‘6’ for HPV and HSV-2 bi-infection and ‘7’ for EBV, HPV and HSV-2 tri-infection), partial correlation was carried out to determine the relationship between the expression of the biomarkers in all participants. Results were presented as mean ± standard error of the mean. Statistical significance was set at *p*-values less than 0.05.

## Results

The number (%) of participants (*N* = 319) in the eight groups were: Group 1 (control group/None; virus negative group) = 81 (25.4%), Group 2 (EBV mono-infection) = 15 (4.7%), Group 3 (HPV mono-infection) = 30 (9.4%), Group 4 (HSV-2 mono-infection) = 67 (21.0%), Group 5 (EBV and HPV bi-infection) = 5 (1.6%), Group 6 (EBV and HSV-2 bi-infection) = 46 (14.4%), Group 7 (HPV and HSV-2 bi-infection) = 22 (6.9%) and Group 8 (EBV, HPV and HSV-2 tri-infection) = 53 (16.6%).

### Expression of oncomirs in viral infection

MiR-21 was significantly overexpressed among participants with EBV mono-infection (*p* = 0.047), HPV mono-infection (*p* < 0.001), and EBV-HPV bi-infection (*p* = 0.015) when compared with the control group (Figure 1a). A statistically significant overexpression of miR-182 among participants with HSV-2 mono-infection (*p* = 0.043), EBV-HSV-2 bi-infection (*p* = 0.002), HPV-HSV-2 bi-infection (*p* = 0.011), and EBV-HPV-HSV-2 tri-infection (*p* = 0.018) was observed when compared with the control group (Figure 1b). A significant miR-155 overexpression among participants with EBV mono-infection was observed when compared with the control group (*p* < 0.001; Figure 1c). miR-200c overexpression in EBV mono-infection (*p* = 0.025) and HPV mono-infection (*p* = 0.003) when compared with the control group was statistically significant (Figure 1d). Expression of both miR-21 (Figure 1a) and miR-200c (Figure 1d) were higher among participants with HPV mono-infection than participants with other viral status (test groups) at *p* = 0.009 and *p* = 0.073, respectively. In terms of expression level, irrespective of viral status, results showed significant direct relationships between miR-21 and miR-200c (*p* < 0.001), between miR-182 and SCCA1 (*p* = 0.051) and between the p53 gene and some miRNAs (miR-21, *p* < 0.001; miR-155, *p* < 0.001; miR-200c, *p* = 0.001). Statistically significant inverse relationships were observed between miR-143 and miR-200c (*p* = 0.025) and between miR-200c and CD4+ T-cell counts (*p* = 0.048; Figures 1d and 3b). In terms of virus status, (infected groups), significant direct relationships were observed between miR-21 and miR-200c (*p* = 0.053), miR-21 and Ki67 (*p* = 0.045), miR-125b and miR-143 (*p* < 0.001), and p53 gene and miR-155 (*p* = 0.039). Significant inverse relationships were also observed between miR-200c and miR-143 (*p* = 0.016), and miR-145 and Ki67 (*p* = 0.001). Insignificant direct relationships were also observed between miR-21 and p53 (*p* = 0.075) and miR-200c and p53 (*p* = 0.330), while an insignificant inverse relationship was observed between the miR-182 and p53 gene (*p* = 0.826).

### Expression of tumour suppressors in viral infection

Higher expression was observed for miR-let-7b in EBV and HPV bi-infection (*p* = 0.315), miR-143 in EBV-HPV-HSV-2 tri-infection (*p* = 0.668), miR-125b in EBV mono-infection (*p* = 0.450) and miR-145 in HSV-2 mono-infection (*p* = 0.002). Lower expression was observed for miR-let-7b in EBV mono-infection, miR-143 in HPV mono-infection and miR-125b in HSV-2 mono-infection (Figure 2a–c), when compared with other viral mono-infections. In terms of expression level, significant direct correlations were observed between the following biomarkers: miR143 and miR-145 (*p* = 0.041), miR-143 and SCCA1 (*p* = 0.009), and miR-143 and miR-125b (*p* = 0.052), irrespective of viral status (Figure 2).

Higher expression of p53 was observed among participants who were seropositive for EBV and HPV mono-infections (*p* = 0.014) but lower among those seropositive for EBV-HPV-HSV-2 tri-infection when compared with those who were seronegative for the three viruses (Figure 3a). The result showed that HSV-2-related infections had lower CD4^+^ T-cell counts (*p* = 0.102; 3b). Higher and lower serum levels of Ki67 were observed in HPV and HSV-2 mono-infections (*p* = 0.208), when compared with those who were seronegative for the three viruses (Figure 3c). Lower serum level of SCCA1 was observed in EBV mono-infection compared with other groups (*p* = 0.005; Figure 3d).

### Expression of biomarkers in viral mono-infection and co-infection

**FIGURE 4:**
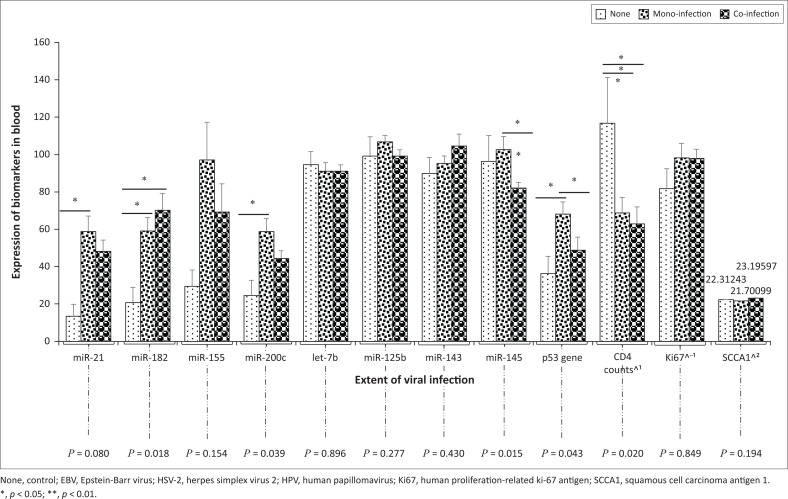
**Expression of biomarkers among individuals with viral mono-infection or co-infection, in the absence of the target viruses, Ogun State, Nigeria, 2018**. Expression of miR-21, miR-155, miR-200c, p53 gene and Ki67 were higher among participants with viral mono-infection than among those with viral co-infection; miR-182 and miR-143 were higher among participants with viral co-infection than among those with mono-infections; miR-let-7b was lower among participants with viral co-infection than among those with mono-infections; miR-125b expression was higher among participants with viral mono-infection than among those with viral co-infection; miR-145 expression was higher among participants with viral mono-infection and lower among those with viral co-infection, CD4^+^ T-cell counts was lower among participants with viral co-infection than among those with viral mono-infection; SCCA1 level was higher among participants with viral co-infection and lower among those with viral mono-infection; when compared with those who had no viral infections.

Data analysis showed a higher expression of the miR-21, miR-155, miR-200c and p53 gene among those with viral mono-infection while a higher expression of miR-182 and lower CD4+ T-cell counts were observed among those with viral co-infection when compared with the control group (Figure 4). The expression of miR-155 and miR-200c among women with a viral co-infection were insignificantly higher when compared with their expression in non-infected women. Individuals with a viral mono-infection had a higher miR-145 expression, while those with viral co-infection had a lower expression of miR-145 when compared with participants who were without any viral infections (*p* = 0.015; Figure 4).

## Discussion

This study estimated the expression of normally downregulated biomarkers (miR-21, miR-155, miR-182, miR-200, p53 gene, Ki67 and SCCA1) and normally upregulated biomarkers (miR-let-7b, miR-125b, miR-143, miR-145 and CD4^+^ T-cells) in viral mono-infection through tri-infection.^6^ Since overexpression of oncomirs and underexpression of tumour suppressors are usually found in all cancers, especially cervical cancer,^17,18,19,20^ this study compared the level of biomarkers in groups 1 to 8 in a bid to identify the biomarkers that are severely affected by viral co-infection. Although higher expression of miR-21 was observed among participants with HPV mono-infection, overexpression of miR-21 among participants with EBV mono-infection suggests that the virus could play an active role in EMT as well. EBV-HPV bi-infection was not associated with higher expressed miR-21 beyond that observed in any of the individual mono-infections, however, it could be inferred that both viruses synergistically dysregulate miR-21. The findings of this study revealed that HSV-2 mono-infection was associated with minimal miR-21-related oncogenic activity when compared with EBV and HPV infection. In other words, this study suggests that HSV-2 mono-infection or its co-infection does not exert substantial oncogenic activity through miR-21 modulation. Additionally, we posit that the oncogenic pathway of each virus varies and may slightly be counteractive during viral co-infection, hence the less expression of miR-21 and miR-155c in viral co-infection. This warrants more study.

In this study, the overexpression of miR-200c in EBV mono-infection, agrees with the findings of Motch et al. which show higher miR-200c expression in EBV-positive tumours than in EBV-negative tumours.^17^ The expression of miR-200c was insignificantly higher in women with viral co-infection than in those who were uninfected. Additionally, the expression of miR200c was significantly higher in women with HPV mono-infection than in women with co-infection, including HSV-2 mono-infection. This also suggests that miR-200c does not play any active roles in oncogenicity associated with viral co-infection. The reason for this is still unknown, but it could be related to differences in the miRNA regulatory pathway or the targeted protein of the investigated viruses. The high expression of miR-200c in HPV mono-infection could be due to its tumourigenic E6/E7 protein.^18^ Again, HSV-2 infection was not associated with substantial dysregulation of miR-200c. Thus, it could be inferred that miR-200c is not directly involved in HSV-2-associated cervical carcinogenesis. In this study, the significant overexpression of miR-155 only in EBV mono-infection suggests that the miRNA expression could be virus dependent and also could be an early indication of EBV infection, immune activation, genetic instability or imminent tumourigenesis.^19,20^ An inverse relationship was observed between miR-155 and miR-let-7b expression in EBV mono-infection. This is in line with the findings of an earlier study which show that the expression of the let-7 family is suppressed in EBV-associated cervical cancer.^21^ Since studies have shown that HPV induces genetic instability and EMT by upregulating miR-182, it could be inferred that HSV-2 plays a similar role in cervical carcinogenesis by upregulating miR-182.^18,22^ The higher upregulation of miR-182 in viral co-infection when compared with viral mono-infection suggests that EBV, HPV and HSV-2 co-infection synergistically modulates the biomarker, possibly through a similar target protein. The triple impact of the viruses also suggests that the rate of EMT could be faster in women with viral co-infection than in women with mono-infection. Again, further studies are required. The findings of the current study also revealed that, although not statistically significant, the expression of ki67 and SCCA1 was relatively higher among participants with a viral infection and that a significant increase in these biomarkers could be indicative of cancer.

Viruses, individually or synergistically, have developed unique mechanisms for hijacking or inhibiting the tumour suppressive functions of wildtype p53.^23^ Since there were direct relationships between the p53 gene, miR-21 and miR-200c in terms of viral infection, the higher expression of the p53 gene (tumour suppressor) in participants with EBV and HPV mono-infections could be an immunologic response to higher miR-21 and miR-200c (oncogenes) among participants with mono-infection. This could be an early indicator of cell cycle dysregulation. This higher expression of the p53 gene in HPV mono-infection was also observed by Zatonski et al.^24^ Continual reduction in its expression or its mutation during viral co-infection due to immune exhaustion, as seen in this study, or underexpression may trigger cervical carcinogenesis. MicroRNA-125b is abundantly expressed in the healthy cervix. It increases shortly after HPV infection and is inactivated or relatively decreased as cancer develops.^25,26^ Decreases in miR-125b expression and the development of cancer may occur due to viral co-infection, especially EBV-HPV-HSV-2 co-presence.

Underexpression of miR-143 and miR-145 has also been reported in cervical cancer, especially in the presence of HPV type 31 E7 oncoprotein.^18,27,28,29^ In this study, while miR-143 overexpression was associated with viral co-infection, miR-145 underexpression was associated with viral co-infection, irrespective of HSV-2 status. This suggests that miR-143 overexpression and miR-145 underexpression could be early indicators of viral co-infection and EMT. It also suggests that EBV and HPV may synergistically and more aggressively promote EMT than either of them could have done unilaterally. Although miR143 and miR145 are tumour suppressors, the divergent expression of both miRNAs in different viral status suggests that they may have dual functions. The latter may be an explanation for the discordant reports on the expression of both biomarkers in cervical cancer.^18,22,30,31^ Furthermore, the higher expression of miR-143 and miR-145, including miR-let-7b and miR-125b, among women with HSV-2 mono-infection when compared with the control group suggests that the miRNAs do not play any significant role in HSV-2-associated oncogenic activity. However, it could be argued that HSV-2 infection contributes to cervical carcinogenesis by reducing CD4^+^ T-cell counts. This is underscored by the finding that a decrease in CD4^+^ T-cell counts by 100 cells/mm^3^ increases the risk of cervical lesions by 13% to 18%.^32^

### Limitations

The expression of miRNAs in a few cases of EBV-HPV bi-infection (*n* = 5) and EBV mono-infection (*n* = 15) as seen in this study may not allow for conclusive generalisation in the investigated population. In other words, a clear and significant impact of EBV-HPV bi-infection and EBV mono-infection on miRNAs may be observed when a larger population is investigated. However, this study could serve as a baseline for future studies. Additionally, the use of conventional reverse transcriptase PCR in this study is a limitation, as expression levels had to be determined by densitometric analysis. We believe that the use of quantitative real-time PCR could have yielded superior results than reverse transcriptase PCR.

### Conclusion

The findings of this study show that while EBV and HPV infections may synergistically target miR-21 and miR-200c, HSV-2 and its related co-infections may target miR-182 and CD4^+^ T-cell activity to initiate EMT. Thus, this study suggests that viral co-presence increases the risk of cervical carcinogenesis by downregulating miR-145, upregulating miR-182 and suppressing CD4^+^ T-cell activity.
